# Extracorporeal Membrane Oxygenation for Fulminant Myocarditis: Increase of Cardiac Enzyme and SOFA Score Is Associated with High Mortality

**DOI:** 10.3390/jcm10071526

**Published:** 2021-04-06

**Authors:** Yun Im Lee, Suryeun Chung, Ji-Hyuk Yang, Kiick Sung, Darae Kim, Jin-Oh Choi, Eun-Seok Jeon, Jeong Hoon Yang, Yang Hyun Cho

**Affiliations:** 1 Department of Internal Medicine, National Cancer Center, Goyang-si 10408, Korea; twirline@gmail.com; 2 Department of Thoracic and Cardiovascular Surgery, Samsung Medical Center, Sungkyunkwan University School of Medicine, Seoul 06351, Korea; suryeun.chung@samsung.com (S.C.); jh1.yang@samsung.com (J.-H.Y.); kiick.sung@samsung.com (K.S.); 3 Division of Cardiology, Department of Medicine, Samsung Medical Center, Sungkyunkwan University School of Medicine, Seoul 06351, Korea; darae0918kim@samsung.com (D.K.); jinoh.choi@samsung.com (J.-O.C.); eunseok.jeon@samsung.com (E.-S.J.); jeonghoon.yang@samsung.com (J.H.Y.); 4 Department of Critical Care Medicine, Samsung Medical Center, Sungkyunkwan University School of Medicine, Seoul 06351, Korea

**Keywords:** fulminant myocarditis, myocarditis, extracorporeal membrane oxygenation

## Abstract

We aimed to evaluate the outcomes of patients with fulminant myocarditis and investigate the factors associated with mortality. This is a retrospective single-center cohort study that included adult and pediatric patients with fulminant myocarditis treated at Samsung Medical Center between September 2004 and December 2019. The primary outcome was in-hospital mortality. Among 100 patients, 71 underwent veno-arterial extracorporeal membrane oxygenation (ECMO) (ECMO group). Comorbidities were not significantly different between the ECMO and non-ECMO groups. Cardiac enzymes, creatinine, and median sequential organ failure assessment (SOFA) score at intensive care unit admission were significantly different between the groups. Twenty patients (28.7%) in the ECMO group and two (6.9%) in the non-ECMO group died in-hospital (*p* = 0.02). The significant risk factors of in-hospital mortality were creatine kinase MB fraction (CK-MB) and SOFA score (*p* = 0.009 and *p* = 0.001, respectively) in the ECMO group. In the receiver-operating characteristic curve analysis, the cutoffs of SOFA score and CK-MB were 12 and 94.74 ng/mL, respectively. The patients with both variables above the cutoffs showed significantly worse outcomes (*p* < 0.001). ECMO can be an effective treatment option for fulminant myocarditis. SOFA score and CK-MB are significant risk factors for in-hospital mortality.

## 1. Introduction

Acute myocarditis is a disease with a broad spectrum of clinical presentations that range from flu-like illness to cardiogenic shock or even death. Particularly, acute myocarditis with hemodynamic instability, so-called fulminant myocarditis, has not yet been well characterized, despite its risk of fatal outcomes. Furthermore, as the utilization of extracorporeal membrane oxygenation (ECMO) has increased, its use in cases of fulminant myocarditis has also increased [1,2]. However, few studies have investigated the use of ECMO support for patients with fulminant myocarditis.

In addition, the risk factors that are associated with outcomes of fulminant myocarditis are not well established. Some studies have reported that poor left ventricular systolic function and arrhythmias were associated with poor clinical outcomes [3,4]. On the other hand, there were several publications that reported that these variables did not contribute to the prognosis of acute myocarditis [5]. 

Thus, we aimed to evaluate the outcomes of patients with fulminant myocarditis, including those who underwent ECMO, for cardiogenic shock or cardiac arrest. Additionally, we also investigated the risk factors associated with in-hospital mortality of patients with fulminant myocarditis.

## 2. Materials and Methods

### 2.1. Study Population

This is a retrospective single-center cohort study. Eligible patients were adult and pediatric patients admitted to the intensive care units (ICUs) of our tertiary hospital (Samsung Medical Center, Seoul, Korea) between September 2004 and December 2019 because of fulminant myocarditis. Patients with non-fulminant myocarditis, bacterial infective endocarditis, primary valvular heart disease, or uncorrected congenital heart disease were excluded.

We reviewed the patients’ electronic medical charts for their baseline characteristics, including comorbidities, ICU management, and clinical outcomes. Our institutional prospective registry for ECMO was also reviewed. The study was approved by the institutional review board of Samsung Medical Center, Sungkyunkwan University (2020-02-11-001, date of approval 18 March 2020). The need for patient consent to participate in the study was waived as we collected data from preexisting medical records.

### 2.2. Definitions and Outcomes

Acute myocarditis is defined by acute symptoms such as chest pain, dyspnea, or unexplained cardiogenic shock with newly developed electrocardiographic features (e.g., atrioventricular block, bundle branch block ST/T wave change, widened QRS complex or ventricular tachycardia, etc.) and elevated troponin T or I. Coronary angiography was performed to rule out coronary artery diseases. In addition, endomyocardial biopsy or cardiac MRI was performed if possible [6,7]. Fulminant myocarditis is defined as acute myocarditis with hemodynamic instability requiring high doses of vasopressors (≥5 μg/(kg·min) dopamine, dobutamine, or other inotropic equivalents) or mechanical circulatory support, despite maximal medical treatment. Acute myocarditis with refractory ventricular tachyarrhythmia or cardiac arrest was also included [8,9,10]. The sequential organ failure assessment (SOFA) score was calculated using the worst values within 24 h from ICU admission [11]. We used the pediatric SOFA score for patients under 18 years old [12]. The primary outcome was in-hospital mortality, while the long-term outcomes included the New York Heart Association (NYHA) class at last follow-up and ejection fraction (%) on last echocardiography.

### 2.3. Statistical Analyses

We reported the descriptive statistics as median (from the 25th to the 75th percentile) and proportion (percentage) for continuous and categorical variables, respectively. We used the Kolmogorov-Smirnov test, the Shapiro-Wilk test, and a histogram to verify the normality of the distribution of the continuous variables. Demographic and clinical differences between the study groups were assessed using the chi-square test, Fisher exact test, Student T test, or Mann-Whitney test, as appropriate. The variables included in the univariable analysis were age, sex, presence of cardiopulmonary resuscitation, initial ejection fraction (%), and utilization of organ support modalities during ICU stay. Variables with *p* values <0.2 in the univariable analysis and considered clinically relevant were included in the multivariable logistic regression. *p* values of <0.05 were considered statistically significant. *P* values were adjusted using Bonferroni correction. We computed odds ratios (ORs) with 95% confidence intervals (CIs). We analyzed the data using IBM SPSS version 25.0 statistical software for Windows (IBM, Armonk, NY, USA), GraphPad Prism 8 (GraphPad Software, San Diego, CA, USA), and MedCalc 19.2.0. (MedCalc Software, Ostend, Belgium).

## 3. Results

### 3.1. Baseline Characteristics

**Figure 1:**
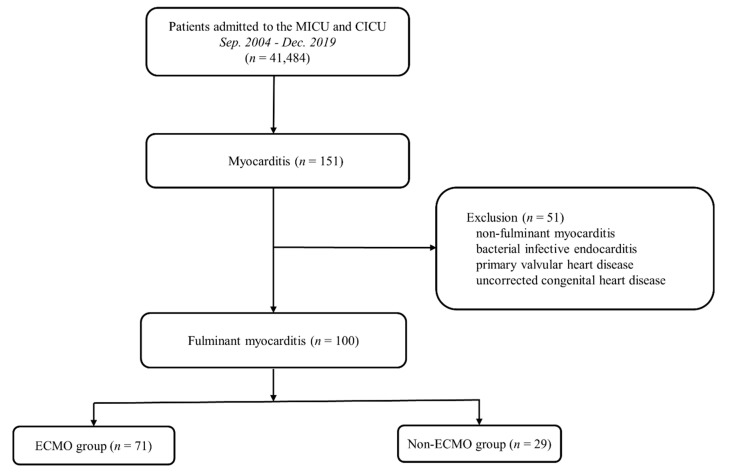
Study flowchart. CICU: cardiac intensive care unit; ECMO: extracorporeal membrane oxygenation; MICU: medical intensive care unit.

From September 2004 to December 2019, 41,484 patients were admitted to the medical and cardiac ICUs of our institution. Among these patients, 151 were diagnosed with acute myocarditis; however, 51 were excluded as they did not meet the inclusion criteria. Thus, 100 patients were included in the study (Figure 1).

Among the 100 patients, 32 (32%) were pediatric patients under 18 years old. Forty-nine patients (49%) had pathologically confirmed myocarditis. Two patients (4%) were diagnosed with giant cell myocarditis and 38 (77.6%) with lymphocytic myocarditis. The median age of the pediatric and adult patients was 4 years (0.25–9.75 years) and 41.5 years (31.25–52 years), respectively. Forty-four patients (44%) were male, and 71 (71%) received ECMO support (ECMO group). Comorbidities, including chronic kidney disease and previous coronary artery disease, were not significantly different between the ECMO and non-ECMO groups. Thirty patients (30%) had cardiac arrest during hospitalization, and 15 (15%) underwent ECMO during cardiopulmonary resuscitation. Most parameters at ICU admission, including white blood cell count, cardiac enzyme levels, creatinine level, lactic acid level, ejection fraction (%), and SOFA score, were significantly different between the two groups. Organ support devices, including mechanical ventilators, dialysis machines, and intra-aortic balloon pumps, were more frequently used in the ECMO group than in the non-ECMO group. In the ECMO group, 10 patients received central veno-arterial ECMO. Among them, four pediatric patients underwent central ECMO at the time of ECMO initiation. The other six adult patients underwent conversion of ECMO configuration from peripheral to central ECMO as the duration of ECMO support lengthened. Twenty-nine patients underwent left ventricular decompression via percutaneous or surgical ways. Details of the patients’ baseline characteristics and ICU data are shown in Table 1.

### 3.2. Clinical Outcomes and Predictors of In-Hospital Mortality

Twenty patients (28.7%) in the ECMO group and two (6.9%) in the non-ECMO group died in-hospital (*p* = 0.02). Eight patients (11.3%) in the ECMO group received heart transplantation or a ventricular assist device during hospitalization. The long-term outcomes of the survivors were also analyzed. The median follow-up duration was 456 days (99–1338 days). Three patients died after discharge, all of whom belonged to the ECMO group. However, the causes of their deaths were not related to myocarditis. One patient died of a hematological malignancy, and another died of pneumonia. The remaining patient died of acute myocardial infarction two years after discharge. In addition, the median NYHA class and ejection fraction (%) of the survivors were normal at the last follow-up and were not significantly different between the two groups (*p* = 0.453 and *p* = 0.059, respectively; Table 2).

**Figure 2:**
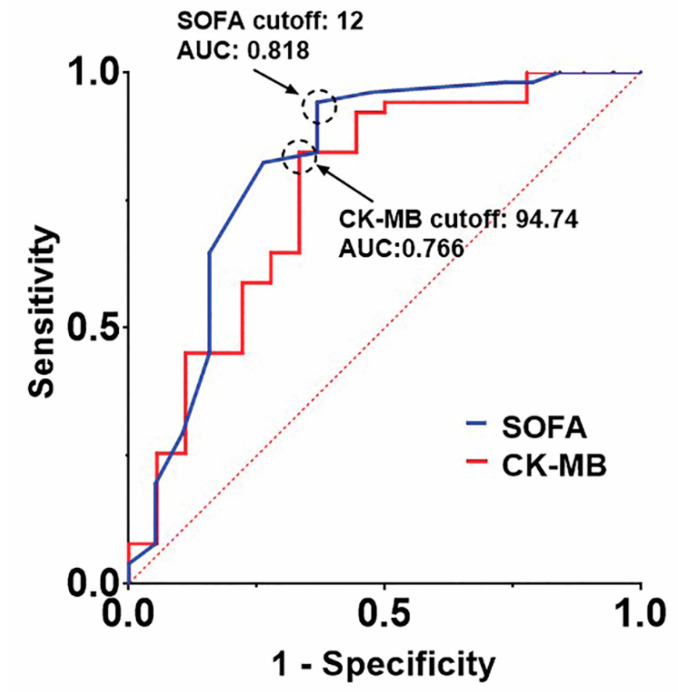
The receiver-operating characteristic curves of the SOFA scores and CK-MB levels in the ECMO group. CK-MB: creatine kinase myocardial band fraction; ECMO: extracorporeal membrane oxygenation; SOFA: sequential organ failure assessment.

**Figure 3:**
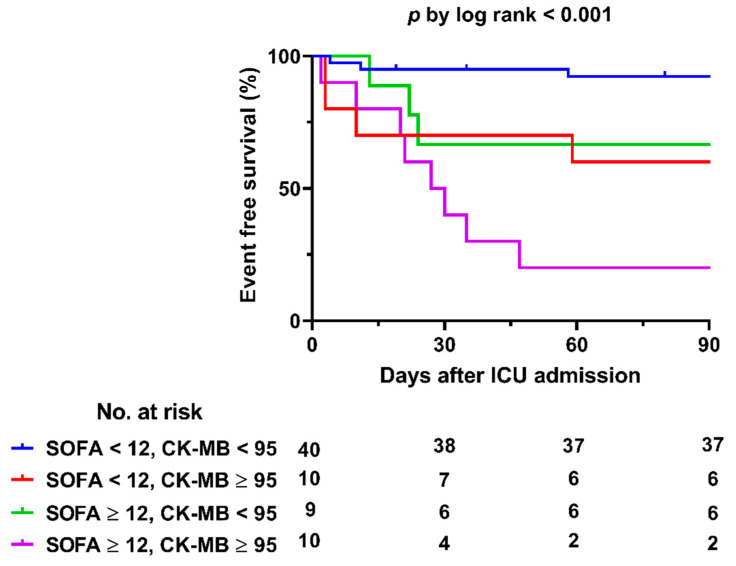
Kaplan-Meier curves of the patients in the ECMO group. CK-MB: creatine kinase MB fraction; ECMO: extracorporeal membrane oxygenation; ICU, intensive care unit; SOFA: sequential organ failure assessment.

In the multivariable analysis, SOFA score was the only independent risk factor of in-hospital mortality (OR, 1.715; 95% CI, 1.304–2.256; *p* < 0.001) in the overall cohort. In the ECMO group, both creatine kinase myocardial band fraction (CK-MB) level and SOFA score were significant risk factors of in-hospital mortality (OR, 1.014; 95% CI, 1.003–1.024; *p* = 0.009 and OR, 1.499; 95% CI, 1.180–1.903; *p* = 0.001, respectively). The results of the univariable and multivariable analyses are shown in Table 3. In the receiver-operating characteristic curve analysis for the ECMO group, the areas under the curve of SOFA score and CK-MB level were 0.818 (cutoff value, 12, sensitivity, 0.941; and specificity, 0.611) and 0.766 (cutoff value, 94.74 ng/mL; sensitivity, 0.843; and specificity 0.667), respectively (Figure 2). The in-hospital mortality rates of the patients with SOFA scores of ≥12 or CK-MB levels of ≥94.74 ng/mL in the ECMO group were both 60%. The patients with both variables higher than the cutoff values showed significantly worse outcomes than those with none or only one (log rank *p* < 0.001; Figure 3).

## 4. Discussion

This study evaluated the outcomes of patients with fulminant myocarditis and the risk factors associated with in-hospital mortality. We found that the SOFA score was a significant risk factor associated with in-hospital mortality from fulminant myocarditis irrespective of ECMO deployment. In the ECMO group, both the SOFA score and CK-MB level were the significant risk factors of in-hospital mortality. Moreover, we found that the late clinical outcomes of the survivors were not significantly different between the ECMO and non-ECMO groups.

Previous studies described the association between organ failure and mortality of fulminant myocarditis [13,14]; however, they did not suggest any measurable variables of mortality. We found that the SOFA score was a significant prognostic factor in both the overall cohort and the ECMO group. A SOFA score of 12 was the optimal cutoff score for hospital survival in the ECMO group. The SOFA score, one of the most validated scoring systems, objectively shows the degree of multiple-organ failure. A high SOFA score suggests a high degree of multiple-organ failure caused by cardiogenic shock in patients with myocarditis. In one study, the SOFA score showed superiority to other scoring systems such as Acute Physiology and Chronic Health Evaluation II score in predicting the mortality of patients with cardiogenic shock [15]. Ferreira et al. described that an initial SOFA score above 11 corresponded to a mortality rate of 95% in critically ill patients [16]. Other researchers also reported that patients with a SOFA score of 12 at ICU admission showed a mortality rate of >50% [17]. Considering the survival rates of patients with myocarditis in the Extracorporeal Life Support Organization registry reports, which range from 50% to 70%, we believe that a SOFA score of 12 is a reasonable criterion [1,2].

The cutoff CK-MB level of 94.74 ng/mL in the present study is almost 20 times the upper normal limit (normal CK-MB range in our hospital: 0–4.87 ng/mL). In previous studies, elevated CK-MB level of >10 times the upper normal limit in the peri-revascularization period was associated with increased mortality in patients with coronary artery disease [18,19,20]. However, no study has described a significant CK-MB criterion in patients with fulminant myocarditis treated with ECMO support. We believe that such a high CK-MB level may represent an extensive myocardial injury caused by both myocardial inflammation and systemic hypoperfusion. Although ECMO can increase the left ventricular afterload, it can also effectively normalize systemic perfusion. The improvement of overall perfusion can enhance myocardial perfusion by increasing blood pressure and decreasing the need for catecholamines. Thus, we propose that for patients with fulminant myocarditis who had an initial SOFA score of 12 and a CK-MB level 20 times the upper normal limit at ICU admission, initiation of ECMO may be reconsidered.

From the perspective of long-term outcomes, some reports delineated that most survivors could sustain their daily lives without critical sequelae [8,21,22,23]. This study revealed similar results. On the other hand, we could not find a significant association between arrhythmias and in-hospital mortality, unlike the study by Sawamura et al. [4]. In their study, the prevalence of ventricular tachyarrhythmia was approximately 16%, which was much lower than that in our study (33%). The prevalence rate of ventricular tachyarrhythmia might have been lower as they excluded patients with severe complications within 24 h after the initiation of ECMO. The prevalence rate of ventricular tachyarrhythmia in patients with fulminant myocarditis rescued with mechanical circulatory support ranges from 3% to over 50% according to previous reports [24,25,26,27,28]. Although the relationship between arrhythmias and mortality in fulminant myocarditis needs further investigation, we believe that refractory malignant arrhythmia strongly relates to irreversible myocardial damage. We had few patients with fulminant myocarditis treated with ECMO support who had continuous asystole or ventricular fibrillation (an additional movie file shows this; see Supplementary Video S1).

This study has a few limitations. First, patients without histopathological confirmation of fulminant myocarditis were included. Second, few patients transferred from the ICUs of other hospitals were included because our institution is a tertiary-care referral center. Third, there is the risk of selection bias that could have affected the results. Fourth, the exact duration of symptoms before patients developed hemodynamic instability was not collected. Fifth, electric-powered catheter venting device, so-called “catheter ventricular assist device”, was not available. Although we liberally used either interventional or surgical left heart venting (25.4%), catheter ventricular assist device may further facilitate the use of left heart decompression and clinical outcome. Finally, this was a retrospective study. Further investigation is needed to corroborate our results.

## 5. Conclusions

Although patients with fulminant myocarditis who required ECMO were in much worse medical conditions than those who did not require ECMO, the early and late outcomes of the former were satisfactory. SOFA score and CK-MB level are significant risk factors that are associated with in-hospital mortality of patients with fulminant myocarditis who were supported with ECMO. Specially, patients with SOFA score above 12 and CK-MB above 94.74 ng/mL showed significantly worse prognosis than others who did not meet the criteria.

## Figures and Tables

**Table 1:** Baseline characteristics of the patients.

	ECMO (*n* = 71)	Non-ECMO (*n* = 29)	*p* -Value
Patient demographics			
Age (year)	34 (19–46)	14 (4–49.5)	0.14
Adult (≥18 year)	41 (31–49) (*n* = 55)	52 (39–56.50) (*n* = 13)	0.069
Pediatric (<18 year)	4 (0–7.75) (*n* = 16)	6 (1.5–10.75) (*n* = 16)	0.364
Gender, male	25 (35.2%)	19 (65.5%)	0.006
BSA (m^2^)	1.60 (1.40–1.69)	1.43 (0.68–1.72)	0.137
Smoking	14 (20%)	3 (10.3%)	0.246
Diabetes mellitus	14 (19.7%)	1 (3.4%)	0.039
Hypertension	15 (21.1%)	2 (6.9%)	0.086
Malignancy	4 (5.6%)	2 (6.9%)	0.809
Dyslipidemia	3 (4.3%)	1 (3.4%)	0.857
Chronic kidney disease ^a^	3 (4.3%)	0 (0%)	0.261
Previous coronary artery diseases ^b^	4 (5.6%)	0 (0%)	0.192
Cardiac arrest	24 (33.8%)	6 (20.7%)	0.194
ECPR ^c^	15 (21.1%)	0 (0%)	NA
Data at ICU admission			
Cardiac enzymes			
Troponin I (ng/mL)	16.96 (3.42–41.70)	4.35 (1.25–21.15)	0.007
CK-MB (ng/mL)	55.76 (23.96–107.36)	24.59 (11.92–68.76)	0.026
NT-proBNP (pg/mL) *	15618 (7582–32,400)	7839 (3223–26,338)	0.068
WBC (×10^3^/μL)	12.79 (9.17–16.92)	9.73 (8.11–13.86)	0.037
CRP (mg/dL)	4.27 (1.32–11.34)	2.71 (0.56–6.51)	0.087
Creatinine (mg/dL)	1.04 (0.80–1.51)	0.62 (0.48–1.04)	<0.001
Lactic acid (mmol/L)	5.09 (2.97–9.08)	2.33 (1.41–3.81)	<0.001
EF (%) at ICU admission	20.0 (15.0–34.0)	40.4 (36.1–58.5)	<0.001
SOFA score ^d^	9	5	<0.001
Documented arrhythmia	63 (90%) (*n* = 70)	23 (79.3%)	0.152
Asystole	5 (7.1%)	0 (0%)	
Brady-arrhythmia ^e^	12 (17.1%)	5 (17.2%)	
Tachy-arrhythmia ^f^	36 (51.4%)	7 (24.1%)	
VT/VF ^g^	30 (42.9%)	3 (10.3%)	
Widened QRS complex ^h^	3 (4.3%)	5 (17.2%)	
Other arrhythmias ^i^	7 (10%)	6 (20.7%)	
Mechanical ventilator	62 (87.3%)	11 (37.9%)	<0.001
CRRT ^j^	27 (38.0%)	1 (3.4%)	<0.001
IABP ^k^	18 (25.4%)	2 (6.9%)	0.036

^a^ Chronic kidney disease is defined as either kidney damage or GFR <60 mL/min/1.73 m^2^ for ≥3 months; ^b^ Previous coronary artery diseases include stable angina, unstable angina and myocardial infarction; ^c^ ECPR is defined by successful veno-arterial extracorporeal membrane oxygenation implantation and pump-on with external chest compression during the index procedure in patients with cardiac arrest; ^d^ Pediatric SOFA score was used for patients under 18 years old; ^e^ Brady-arrhythmia includes bradycardia in children, high-degree atrioventricular block, and third degree atrioventricular block; ^f^ Tachy-arrhythmia includes atrial fibrillation with rapid ventricular response, supraventricular tachycardia, ventricular tachycardia and ventricular fibrillation; ^g^ Ventricular tachycardia (VT) is a cardiac arrhythmia of ≥3 consecutive complexes originating in the ventricles at a rate over 100 beat per minute. Ventricular fibrillation (VF) is a rapid, grossly irregular electrical activity with marked variability in electrocardiographic waveform and ventricular rate usually over 300 beat per minute; ^h^ Widened QRS complex is defined as QRS complex ≥120 milliseconds; ^i^ Other arrhythmias include Q wave, ST depression, ST elevation, first atrioventricular block and second atrioventricular block Mobitz type I with normal heart rate; ^j^ Continuous renal replacement therapy (CRRT) is an extracorporeal blood purification therapy intended to substitute for impaired renal function over an extended period of time; ^k^ Intra-aortic balloon pump counterpulsation (IABP) is a mechanical hemodynamic support device that assists the heart indirectly by decreasing the afterload and augments diastolic aortic pressure; * Maximum value of NT-proBNP measurable in our hospital is 35,000 pg/mL. BSA, body surface area; BUN, blood urea nitrogen; CK-MB, creatine kinase MB fraction; CPR, cardiopulmonary resuscitation; CRP, C-reactive protein; CRRT, continuous renal replacement therapy; ECMO, extracorporeal membrane oxygenation; ECPR, extracorporeal cardiopulmonary resuscitation; EF, ejection fraction; IABP, intra-aortic balloon pump counter-pulsation; ICU, intensive care unit; NA, not applicable; NT-proBNP, N-terminal pro-brain natriuretic peptide; VT, ventricular tachycardia; VF, ventricular fibrillation; WBC, white blood cell. Reported are *n* (%) for categorical variables and median (1 IQR~3 IQR) for continuous variables.

**Table 2:** Clinical outcomes.

	All (*n* = 100)	ECMO (*n* = 71)	Non-ECMO (*n* = 29)	*p*-Value
In-hospital mortality	22 (22%)	20 (28.2%)	2 (6.9%)	0.020
Proportion of heart transplantation/VAD	8 (8%)	8 (11.3%)	0 (0%)	0.101
Long-term outcomes (*n* = 78)				
Death after hospital discharge	3 (3.8%)	3 (5.9%)	0 (0%)	0.547
Median NYHA class of the survivors	1	1	1	0.453
EF (%) at last echocardiography during follow-up	61.7 (56–66.8)	60 (52.5–65)	63 (60.1–67.8)	0.059

ECMO, extracorporeal membrane oxygenation; EF, ejection fraction; NYHA, New York Heart Association; VAD, ventricular assist device.

**Table 3:** Multivariable analysis of in-hospital mortality.

	Univariable Analysis		Multivariable Analysis	
	OR (95% CI)	*p*-Value	OR (95% CI)	*p*-Value
Overall cohort				
Age	1 (0.955–1.048)	0.985		
Gender, male	0.608 (0.094–3.947)	0.602		
Deployment of ECMO	0.563 (0.022–14.386)	0.729		
CRRT	2.889 (0.511–16.332)	0.230		
Cardiac arrest	1.140 (0.163–7.942)	0.895		
EF (%) at ICU admission	0.976 (0.919–1.036)	0.419		
CRP	1.003 (0.871–1.154)	0.972		
Lactic acid	1.106 (0.905–1.352)	0.323		
CK-MB	1.006 (0.997–1.015)	0.212	1.006 (0.998–1.013)	0.139
SOFA score	1.480 (1.044–2.098)	0.028	1.715 (1.304–2.256)	<0.001
ECMO group				
Age	0.991 (0.950–1.034)	0.678		
Gender, male	0.334 (0.055–2.021)	0.233		
CRRT	2.543 (0.555–11.661)	0.230		
Cardiac arrest	1.028 (0.169–6.235)	0.976		
CK-MB	1.013 (1.003–1.023)	0.011	1.014 (1.003–1.024)	0.009
SOFA score	1.492 (1.089–2.046)	0.013	1.499 (1.180–1.903)	0.001

CK-MB, creatine kinase myocardial band fraction; CRP, C-reactive protein; CRRT, continuous renal replacement therapy; ECMO, extracorporeal membrane oxygenation; EF, ejection fraction; ICU, intensive care unit; SOFA, sequential organ failure assessment.

## Supplementary Materials

The following are available online at https://www.mdpi.com/article/10.3390/jcm10071526/s1, Video S1: The video shows the vital signs of a patient that were supported with ECMO, because of sustained ventricular fibrillation caused by fulminant myocarditis. ECMO, extracorporeal membrane oxygenation.

## Abbreviations

CK-MB: Creatine kinase MB fraction; ECMO: Extracorporeal membrane oxygenation; ICU: Intensive care unit; NYHA: New York Heart Association; SOFA: Sequential organ failure assessment.
